# Leveraging Machine Learning to Identify Subgroups of Misclassified Patients in the Emergency Department: Multicenter Proof-of-Concept Study

**DOI:** 10.2196/56382

**Published:** 2024-12-31

**Authors:** Sage Wyatt, Dagfinn Lunde Markussen, Mounir Haizoune, Anders Strand Vestbø, Yeneabeba Tilahun Sima, Maria Ilene Sandboe, Marcus Landschulze, Hauke Bartsch, Christopher Martin Sauer

**Affiliations:** 1 Department of Global Public Health Faculty of Medicine University of Bergen Bergen Norway; 2 Department of Emergency Medicine Haukeland University Hospital Bergen Norway; 3 Department of Clinical Science Faculty of Medicine University of Bergen Bergen Norway; 4 Helse Vest IKT Bergen Norway; 5 Department of Research and Development Haukeland University Hospital Bergen Norway; 6 Faculty of Medicine and Health Sciences Norwegian University of Science and Technology Trondheim Norway; 7 Department of Computer Science, Electrical Engineering and Mathematical Sciences Western Norway University of Applied Sciences (HVL) Bergen Norway; 8 Mohn Medical Imaging and Visualization Centre Department of Radiology Haukeland University Hospital Bergen Norway; 9 Institute for Artificial Intelligence in Medicine University Hospital Essen Essen Germany; 10 MIT Critical Data Massachusetts Institute of Technology Boston, MA United States; 11 Department of Hematology and Stem Cell Transplantation University Hospital Essen Essen Germany

**Keywords:** emergency department, triage, machine learning, real world evidence, random forest, classification, subgroup, misclassification, patient, multi-center, proof-of-concept, hospital, clinical feature, Norway, retrospective, cohort study, electronic health system, electronic health record

## Abstract

**Background:**

Hospitals use triage systems to prioritize the needs of patients within available resources. Misclassification of a patient can lead to either adverse outcomes in a patient who did not receive appropriate care in the case of undertriage or a waste of hospital resources in the case of overtriage. Recent advances in machine learning algorithms allow for the quantification of variables important to under- and overtriage.

**Objective:**

This study aimed to identify clinical features most strongly associated with triage misclassification using a machine learning classification model to capture nonlinear relationships.

**Methods:**

Multicenter retrospective cohort data from 2 big regional hospitals in Norway were extracted. The South African Triage System is used at Bergen University Hospital, and the Rapid Emergency Triage and Treatment System is used at Trondheim University Hospital. Variables included triage score, age, sex, arrival time, subject area affiliation, reason for emergency department contact, discharge location, level of care, and time of death were retrieved. Random forest classification models were used to identify features with the strongest association with overtriage and undertriage in clinical practice in Bergen and Trondheim. We reported variable importance as SHAP (SHapley Additive exPlanations)-values.

**Results:**

We collected data on 205,488 patient records from Bergen University Hospital and 304,997 patient records from Trondheim University Hospital. Overall, overtriage was very uncommon at both hospitals (all <0.1%), with undertriage differing between both locations, with 0.8% at Bergen and 0.2% at Trondheim University Hospital. Demographics were similar for both hospitals. However, the percentage given a high-priority triage score (red or orange) was higher in Bergen (24%) compared with 9% in Trondheim. The clinical referral department was found to be the variable with the strongest association with undertriage (mean SHAP +0.62 and +0.37 for Bergen and Trondheim, respectively).

**Conclusions:**

We identified subgroups of patients consistently undertriaged using 2 common triage systems. While the importance of clinical patient characteristics to triage misclassification varies by triage system and location, we found consistent evidence between the two locations that the clinical referral department is the most important variable associated with triage misclassification. Replication of this approach at other centers could help to further improve triage scoring systems and improve patient care worldwide.

## Introduction

A triage system is a standardized system for rapid patient decision-making used in emergency departments (EDs) worldwide. Most systems classify patients into different emergency levels based on symptoms and clinical signs [1]. Triage systems are frequently established on the basis of expert opinion and may not consistently undergo validation [2]. Preventing triage misclassification is the main objective of triage. Overprioritizing patients with mild conditions is not the best distribution of limited hospital resources, whereas underprioritizing severe cases can detrimentally impact patient outcomes.

Validation of a triage system can be challenging without a gold-standard assessment of patient urgency. Previous studies have used patient mortality and admission to intensive care units (ICUs) as measures of high urgency and discharge from ED as low urgency [1,3]. All triage systems aim to classify patients by urgency of the condition and availability of resources, but they may vary by the importance of clinical criteria like pain and definitions of time to care for patients classified as urgent. The sensitivity and specificity of different triage systems vary greatly. Most perform moderately well in identifying high-urgency patients (sensitivity between 58% and 100%) but perform significantly worse in identifying low-urgency patients (sensitivity between 8% and 70%). The literature on the validity of triage systems has been limited so far, and no triage system has been identified as clearly outperforming others [1,4]. Triage systems also have variable performance across contexts, reasons for presenting to the ED, patient age, and patient race [5-7].

In Norway, no triage system is used consistently nationwide, with different regions either using the Manchester Triage System (MTS) [8], the Rapid Emergency Triage and Treatment System (RETTS) [9], or a modified version of the South African Triage Scale (SATS) [10]. MTS is among the best-studied systems worldwide, and there is much more limited evaluation of RETTS and SATS, especially considering what factors influence their performance [1,3,4].

So far, most studies evaluating triage systems have relied on domain knowledge to evaluate the importance of factors contributing to triage misclassification [1,4]. While research driven by qualitative insights and posterior probabilities is important, it may also be valuable to evaluate the importance of variables without prior assumptions. In this study, we validated the performance of the modified SATS and RETTS used at Bergen and Trondheim University Hospital and aimed to identify patient clusters that are misclassified using these systems. To capture nonlinear relationships, we used various machine learning (ML) methods and used SHAP-values to establish feature importance.

## Methods

### Data Source

We conducted a multicenter retrospective cohort study at the main ED at Haukeland University Hospital, Bergen, and St. Olav’s University Hospital in Trondheim, Norway. Haukeland University Hospital functions as a referral center for about a million inhabitants in the Bergen metropolitan area [10] and had an annual ED admission ranging from 33,000 to 38,000 during the study period. The ED manages patients with medical, surgical, and neurologic conditions, excluding children with medical issues and pregnant women with obstetric conditions who are treated elsewhere. For the period between 2013 and 2017, comprehensive data on all patients treated in the ED was gathered. This data encompasses administrative details like time of admission, department, and source of admission, as well as clinical information such as age, sex, and triage level. The data collection was conducted using the electronic health system used in the ED (Akuttdatabasen, Helse-Vest IKT, version 1.5.5., Stavanger).

St. Olav’s, a tertiary medical center, serves as the primary health care facility for a local population of 300,000 residents and functions as the regional hospital for the Trondheim metropolitan area in central Norway, catering to over 700,000 residents. The hospital manages around 22,000 emergency department admissions annually [11]. Data from all patient contacts in the emergency department between 2012 and 2022 were extracted. The extraction was based on the ED’s patient database (Akuttdatabasen, Helse Vest IKT, version 1.5.5, Stavanger, Norway). Following this, the extracted data was linked to patient administrative hospital data, with no exclusions made for patient contacts. Variables with incomplete registration or linkage between databases were omitted from individual variable results but retained in the overall dataset. Information encompassing age, sex, arrival time, subject area affiliation, reason for contact, discharge location, level of care, and time of death was accessed in the database. This study received approval from the data protection officer (ESA-no 16/9114).

### Missing Data

Patient information was collected prospectively at both hospitals, and we received access to the data in 2022. The Bergen dataset included all patients who presented at the ED from January 2012 through September 2017, while the Trondheim dataset encompassed patients presenting to the ED from 2012 to 2022. Data in Bergen could not be accessed after 2017 due to changes in the data storage protocol. For both datasets, we excluded patients with missing national identity numbers and no triage scores. For missing categorical data, we included a category “missing” in the model.

### Triage Definitions

The SATS protocol (Bergen) categorizes patients into triage levels: 1 is red (emergency), 2 is orange (very urgent), 3 is yellow (urgent), 4 is green (not urgent), or 5 is blue (can wait). The physician should assess the patient immediately if triaged to level red, within 10 minutes to level orange, within 60 minutes to level yellow, and within 120 minutes to level green [1]. The RETTS protocol (Trondheim) also categorizes patients into triage levels 1 to 5 but has different recommended times until care for each level: immediately for level red, 20 minutes for level orange, 60 minutes for level yellow, and 240 minutes for level green [12]. Blue triage encompasses patients arriving for administrative reasons or scheduled visits in both systems.

### Outcome Definition

The composite endpoint used to define high acuity was defined as (1) death within 24 hours after presentation to the emergency room, (2) transfer to the ICU from the ED, or (3) transfer to the surgical operating theater or for coronary angiography directly from the ED. The composite of these outcomes is hereafter referred to as “severe illness.” Discharge from ED (ie, patients not admitted to the hospital) was the reference standard for low acuity. Undertriage was defined as a patient who died or was admitted to the ICU within 24 hours of presenting to the ED and was given a triage score of level 3, 4, or 5. Overtriage was defined as a patient who was discharged from the ED and was given a triage score of 1 or 2.

### Classification Model

Separate classification models were built to identify variables associated with either under- or overtriage. The 2 subgroups consisting of patients with high or low acuity conditions were used as the training and test dataset, where the target variable was defined as undertriage (yes or no) or overtriage (yes or no), respectively. The models with the higher area under the curve score were used, a Random Forest for the Trondheim dataset and XGBM classifier for the Bergen dataset. The best-performing model was evaluated using a 5-fold cross-validation approach and GridSearch for parameter optimization. To handle the unbalanced data problem, SMOTE (synthetic minority oversampling technique) oversampling was applied to the data, and adjusted class weight parameters of the algorithm (ie, class weight for LR/RF/DT, scale_pos_weight for XGB) were applied to the models. The resulting models were then scored on the test data (30% of the data), and a receiver operating characteristic (ROC) curve score was computed. The feature importance and contributions were analyzed by calculating the SHAP (SHapley Additive exPlanations)-values [13].

### Ethical Considerations

Approval for this study and a waiver of written informed consent was obtained from the Regional Committee for Medical and Health Research Ethics in Western Norway for Bergen University Hospital (case number 2018/2128). At Trondheim University Hospital, this study was classified as a quality assurance study, and a waiver from the Regional Committee for Medical and Health Research Ethics was granted (2016/1813/REK).

## Results

### Overview

Records for 205,488 patients from Bergen University Hospital and 304,997 records from Trondheim University Hospital were included in the final analysis (Table 1). There were few demographic differences between Bergen and Trondheim University Hospital, though a greater percentage of patients in Bergen (24.1%) were given low-priority triage scores than patients in Trondheim (9%, Table 1). Undertriage occurred in 1579 patients in Bergen and 736 patients in Trondheim, while overtriage was observed in 7 patients in Bergen and 22 patients in Trondheim. Missing information was overall low, with a maximum of 2.7% for age in the Bergen dataset.

**Table 1:** Demographic and clinical characteristics of the patients included in the final cohort.

		Bergen University Hospital (N=205,488), n (%)	Trondheim University Hospital (N=304,997), n (%)
**Age groups**			
	<18 years old	14,307 (7)	7362 (2.5)
	18-65 years old	104,634 (50.9)	155,233 (52.3)
	>65 years old	86,321 (42)	134,268 (45.2)
	Missing	226 (0.1)	8134 (2.7)
**Sex**			
	Female	97,535 (47.5)	149,111 (48.9)
	Male	107,945 (52.5)	148,353 (48.6)
	Missing	8 (<0.1)	7533 (2.5)
**Day of week**			
	Weekday	145,952 (7)	217,909 (71.4)
	Weekend	59,535 (29)	87,088 (28.6)
	Missing	1 (<0.1)	0 (0)
**Time of day**			
	Early morning (4:00 to 7:59)	10,781 (5.2)	14,272 (4.7)
	Morning (8:00 to 11:59)	40,242 (19.6)	57,988 (19)
	Noon (12:00 to 15:59)	61,815 (30.1)	97,621 (32)
	Evening (16:00 to 19:59)	44,766 (21.8)	61,456 (20.1)
	Night (20:00 to 23:59)	32,190 (15.7)	46,972 (15.4)
	Late night (00:00 to 3:59)	15,693 (7.6)	26,688 (8.8)
	Missing	1 (<0.1)	0 (0)
**Clinical referral department**			
	Internal Medicine	97,058 (47.2)	169,619 (55.6)
	Surgical	55,491 (27)	78,825 (25.8)
	Others	52,939 (25.8)	56,553 (18.5)
	Missing	0 (0)	0 (0)
**Triage score**			
	Low (1-2, Blue or Green)	49,583 (24.1)	27,300 (9)
	High (3-5, Yellow, Orange, or Red)	155,904 (75.9)	276,933 (90.8)
	Missing	1 (<0.1)	764 (0.3)
**Status at 24 hours**			
	Alive	204,416 (99.5)	303,483 (99.5)
	Deceased	1072 (0.5)	1514 (0.5)
	Missing	0 (0)	0 (0)

### Model Performance

**Figure 1:**
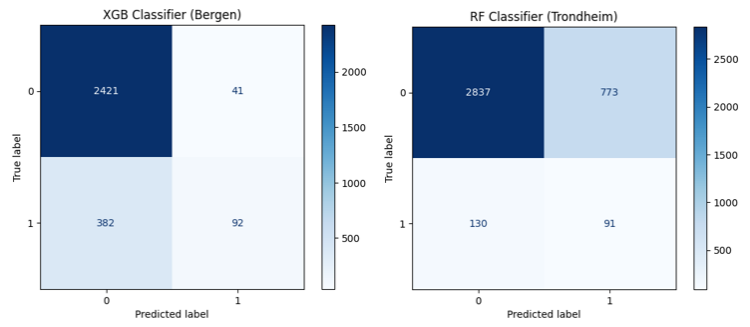
Undertriage classifier confusion matrix for the Bergen University Hospital XGB classifier (left) and Trondheim University Hospital RF classifier (right). XGB: XGBoost; RF: random forest.

**Figure 2:**
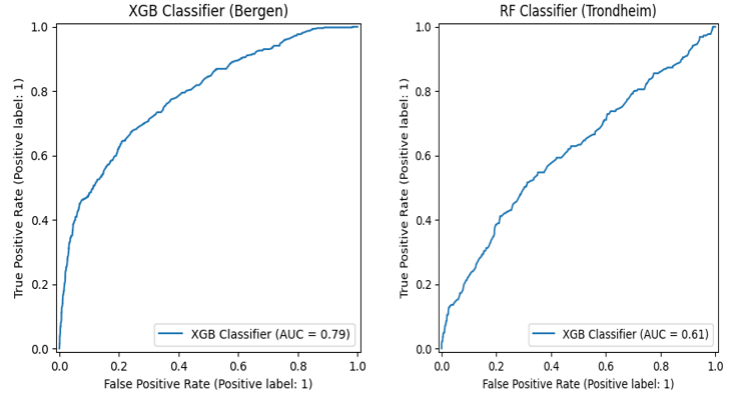
Undertriage classifier area under the receiver operator curve for XGB with Bergen University Data (left) and RF for Trondheim University data (right). XGB: XGBoost; RF: random forest.

All 4 considered statistic models performed similarly for a specific dataset, and little differences between SMOTE and class weight parameter adjustment were found (Table 2). The random forest model was chosen for Bergen University Hospital, and the XGBoost classifier for Trondheim University Hospital. Model performance for undertriage was overall lower for Trondheim University Hospital due to a high number of patients receiving high triage scores but having nonsevere outcomes (Figure 1). ROC curves for the chosen models are provided in Figure 2.

**Table 2:** Undertriage classifier ROC^a^ scores. XGBoost was chosen as the final model for Bergen and Random Forest for Trondheim University Hospital.

	Bergen University Hospital		Trondheim University Hospital	
	SMOTE^b^	Class weight parameter	SMOTE	Class weight parameter
Logistic regression (LR)	0.78	0.78	0.60	0.61
Random Forest (RF)	0.78	0.78	0.59	0.61
XGB Classifier (XGB)	0.75	0.79	0.53	0.60
DecisionTree Classifier	0.77	0.77	0.53	0.60

^a^ROC: receiver operating characteristic.

^b^SMOTE: synthetic minority oversampling technique.

### Undertriage

**Figure 3:**
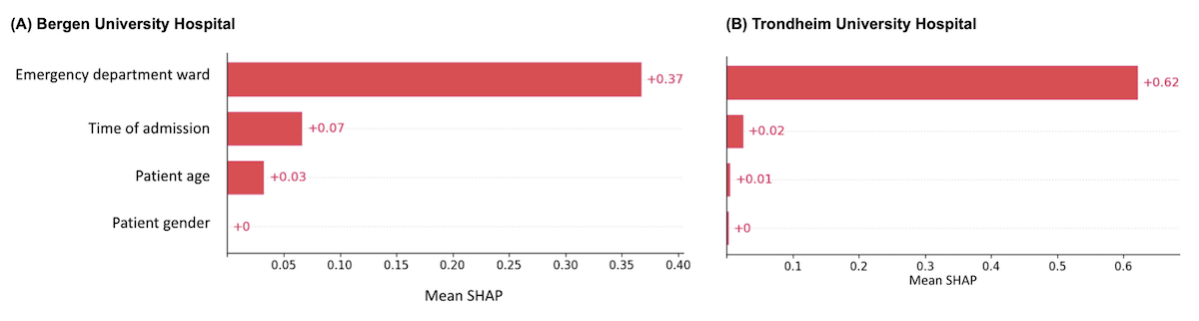
SHapley Additive exPlanations (SHAP)-values for undertriage in the Bergen (A) and Trondheim (B) classification model.

In both the Bergen and Trondheim datasets, the most influential features associated with undertriage were the clinical referral department, time of day of admission, and patient age (Figure 3). The clinical referral department was the only statistically significant variable in the model for the Bergen dataset, while in Trondheim, patient age and time of day were also associated with undertriage (Figures 5 and 6). A bee plot showing all variables included in the models is provided in the supplementary figure (Multimedia Appendices 1 and 2).

**Figure 4:**
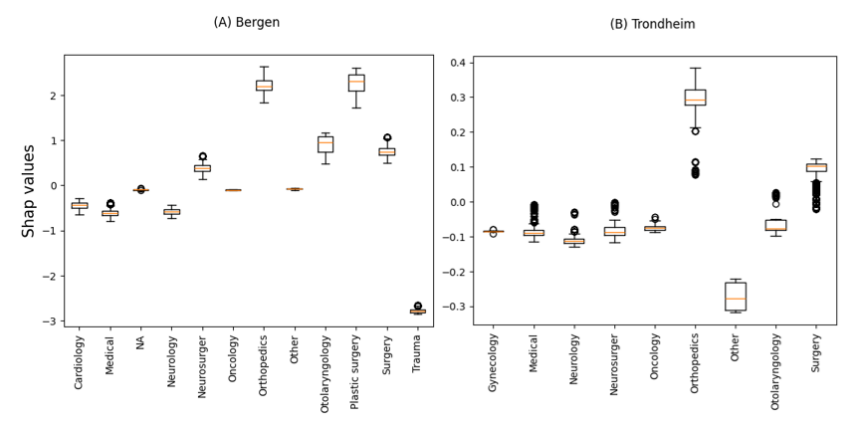
SHapley Additive exPlanations (SHAP)-values for undertriage by clinical assignment category for the Bergen (A) and Trondheim (B) data set. NA: not available.

In the Bergen dataset, orthopedics and plastic surgery clinical assignment categories (Figure 4A) show a shift towards higher SHAP-values, indicating a higher probability of undertriage, while the trauma category shows a shift toward lower SHAP-values, indicating a higher probability of correct triage. There was no specific category of patient age or time of day that was important to the model.

In the Trondheim dataset clinical referral departments such as orthopedics (Figure 4B) and late-night admission time (Figure 6B) have higher SHAP-values indicating a higher probability of undertriage, while patients aged 0 to 17 (Figure 7B) and admission times in the morning showed a trend towards lower SHAP-values indicating a higher probability of correct triage.

**Figure 5:**
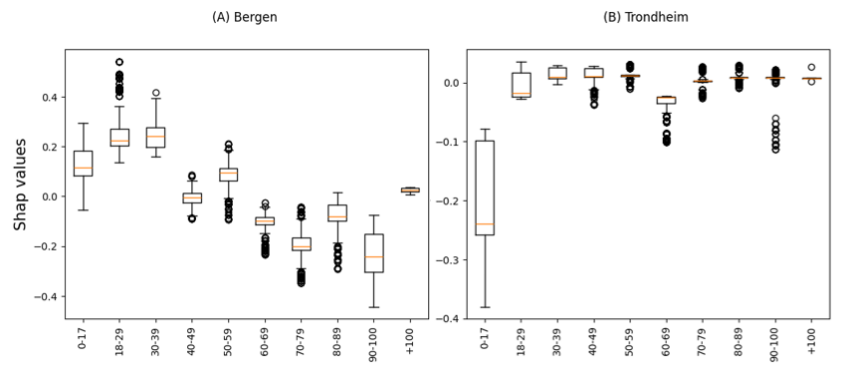
SHapley Additive exPlanations (SHAP)-values for undertriage by age group in the Bergen (A) and Trondheim (B) data set.

**Figure 6:**
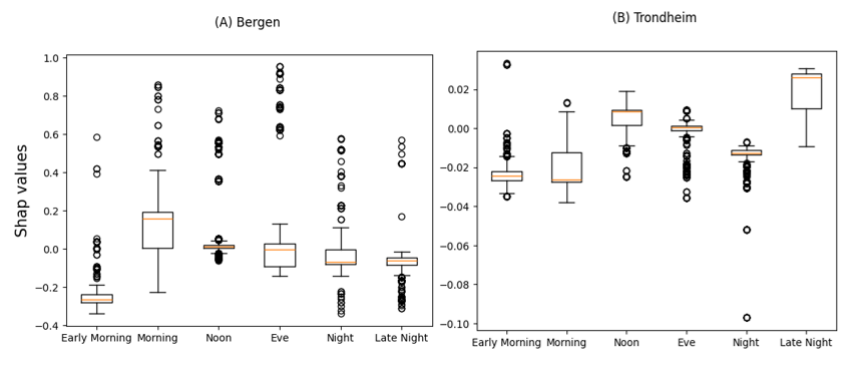
SHapley Additive exPlanations (SHAP)-values for undertriage by time of admission in the Bergen (A) and Trondheim (B) data set.

**Figure 7:**
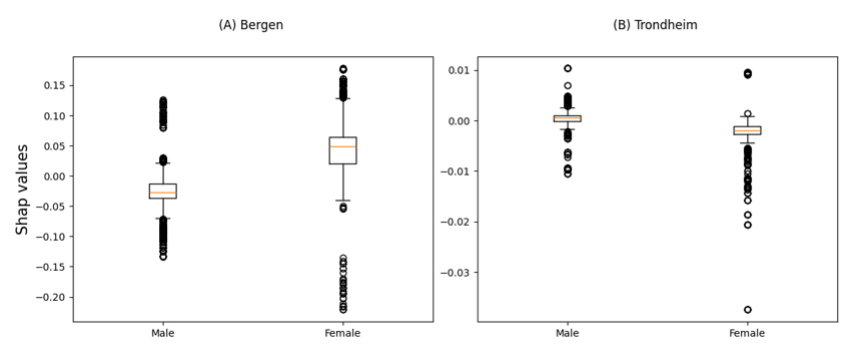
SHapley Additive exPlanations (SHAP)-values for undertriage by sex in the Bergen (A) and Trondheim (B) data set.

### Overtriage

Overtriage was a rare event, only occurring in a total of 29 patients. Results for these exploratory analyses are available from Multimedia Appendix 3.

## Discussion

### Principal Findings

Our study provides a proof of concept of how ML can be leveraged to identify over- and undertriaged patients in the emergency room. As both hospitals function as regional medical centers for surrounding rural areas, they are at high risk of receiving overtriaged transfer patients from smaller regional hospitals without the confidence for definitive treatment [14]. However, the proportion of overtriage was very low (<0.1%), while we found a significant proportion of undertriage (0.2%-0.8%). Improvement of undertriage may, therefore, benefit patients and prevent adverse consequences. However, the increased frequency of undertriage within the orthopedic and surgical emergency groups could also be attributed to the specific definition of high urgency we used. This definition, encompassing factors like direct transfer to the surgical operating theater, aligns with cases requiring prompt surgical intervention but not necessarily immediate life-saving measures, for example, in case of ankle fractures triggering prompt transfers to surgical theaters. In Trondheim, the association between lower age and undertriage is consistent with previous literature on undertriage in children. Most triage systems are refined to adult standards, and triage systems for children can be inaccurate when predicting morbidity and mortality [15].

For overtriaged patients, there was some evidence of the increased frequency of overtriage by the time of day and sex. Both factors can be indicators of health care worker mood and attitudes, and be reflective of biases within the hospital systems [16,17]. However, due to the low number of overtriaged patients (<0.1%), results carry high statistical uncertainty, and the results presented in Multimedia Appendix 3 should be considered explorative only.

### New Applications of ML Methodology

ML is a topic of growing interest in the field of health care, and the number of articles published about ML, specifically in diagnostics, has steadily increased annually since 2000 [18]. ML offers advantages over many conventional statistical methods because they can model nonlinear associations. Previous research considering performance measures only suggests that random forest performs better than the more conventional method of logistic regression in most datasets [19]. Statistical interactions and model multiplicity can also be better captured by ML methods than conventional methods. For optimal usage, appropriate methods must be tailored to the specific research context, and common pitfalls need to be avoided [20].

In this setting, ML methods allowed us to consider the importance of many complex factors that impact triage classification simultaneously, providing more nuanced results than conventional methods. A previous study using conventional methods in Bergen found that overtriage was most prevalent in patients younger than 18 years [21]. Yet the automated variable selection based on SHAP-values in this study revealed that age might not be the most important characteristic contributing to overtriage in Bergen, as might have been an assumption in a study directed by domain knowledge. Instead, with ML methods, we identified that the clinical referral department and *ICD* (*International Classification of Diseases*) diagnostic codes are more important factors associated with overtriage in the Bergen dataset. Our results are in agreement with conventional methods in undertriage, which both showed the triage system as having lower sensitivity to surgical patients than other patients [21].

### Differences Between Triage Systems

The differences seen in overtriage between the Trondheim and Bergen dataset might be due to the usage of different triage systems, as demonstrated in a recent Swedish study [22]. The difference in health system and context between the two Norwegian hospitals is likely not substantial. In the context of health systems, there is a need for more external validation of ML methods against conventional methods in diverse contexts and data [23,24].

Even between 2 hospitals with similar populations and health systems, we could not generalize characteristics important to triage misclassification between the two triage systems. All triage systems are subject to misclassification, but information about the strengths of each system is necessary to create new systems with greater validity. More research is needed in the future about triage systems and new applications of ML methods, such as automated triage classification systems [25]. Identifying which patient groups are at risk of misclassification is a crucial step to reduce health care resource waste and enhance patient safety. Furthermore, this may help to address and reduce health biases.

### Strengths and Limitations

The strengths of this study include large, high-quality data from two different emergency department datasets. Our findings are novel in that we focused our analysis on a comparison in misclassification between two triage systems rather than in the isolated context of one hospital. We are the first to use ML methods to investigate characteristics contributing to misclassification in Norway, yet our approach is largely generalizable beyond Norway and may trigger additional studies investigating the undertriage of patient subgroups.

One limitation of this study pertains to the temporal context of our data acquisition. In 2017, Haukeland Hospital underwent a significant transformation in its computer system used for triage data recording. This shift led to the availability of only aggregated data, devoid of the granular characteristics of individual patient visits that could be effectively linked to other data sources. This transition restricted our ability to work with individual-level data and prompted us to work with somewhat older data for this specific hospital. Despite this challenge, we maintain that the data used remains valid and representative of the patient population that frequents the emergency department. While the more recent transition to aggregated data may have limited our analysis scope, the fundamental characteristics of the patient population, their triage experiences, and the overarching triage dynamics are captured faithfully in our study.

A critical factor in the successful application of ML to ED triage systems is the availability of granular, detailed data. The precision and effectiveness of ML algorithms are directly influenced by the depth and breadth of the data they process. In the context of ED triage, this means having access to comprehensive patient data, including specific reasons for ED visits, presenting symptoms, and vital clinical parameters like blood pressure, heart rate, and respiratory rate. Such detailed data enables the development of more accurate and nuanced ML models capable of making informed triage decisions that closely align with the complexities of real-world clinical scenarios. In addition, granular data is essential in identifying and addressing potential biases inherent in ML models, ensuring that these systems are equitable and effective for diverse patient populations [26].

It is worth acknowledging that our dataset had certain limitations in terms of clinical parameters. While we had access to fundamental data like sex, age, and clinical referral department, our dataset lacked detailed information on the specific reason for ED presentation, presenting symptoms, and clinical parameters like blood pressure, heart rate, or respiratory rate. These clinical details play a pivotal role in the accurate assessment and classification of patient urgency. The absence of these granular clinical parameters may have influenced the precision of our analysis and the generalizability of our findings to a more comprehensive clinical context. Future research would benefit from access to a more comprehensive dataset encompassing these key clinical parameters. As for the risk of inclusion bias, we believe that due to Norway’s highly inclusive and public health care system, this is overall low. However, the underrepresentation of underserved minorities cannot be finally excluded.

The study’s insights underscore the importance of updated and accessible data for robust research in health care. The shift to aggregated data in one of the hospitals due to computer system changes in 2017 serves as a reminder of the dynamic nature of data availability. The ability to work with up-to-date and comprehensive datasets, coupled with the capacity to integrate data from diverse sources, is pivotal in enabling a holistic understanding of complex health care scenarios like emergency department triage.

### Conclusions

In conclusion, we provide a machine-learning framework to identify subgroups of patients that are undertriaged using two common triage scoring systems. Combining clinical knowledge, routinely collected clinical data, and ML can improve patient care and the efficacy of health care delivery. As we move forward, the emphasis on data access, integration, and real-time updating becomes ever more paramount in advancing clinical care.

## Appendix

Multimedia Appendix 1 Beeplot showing the SHAP (SHapley Additive exPlanations)-values for all included variables at Bergen (A) und Trondheim University Hospital (B).

Multimedia Appendix 2 Beeplot showing the SHAP (SHapley Additive exPlanations)-values for all included variables at Bergen (A) und Trondheim University Hospital (B).

Multimedia Appendix 3 Results for the exploratory findings on overtriage for the Bergen and Trondheim data sets.

## Abbreviations

ED: emergency department
ICD: International Classification of Diseases
ICU: intensive care units
ML: machine learning
MTS: Manchester Triage System
RETTS: Rapid Emergency Triage and Treatment System
ROC: receiver operating characteristic
SATS: South African Triage Scale
SHAP: SHapley Additive exPlanations
SMOTE: synthetic minority oversampling technique

## Data Availability

The code underlying these analyses is available from [27]. The datasets generated during and/or analyzed during this study are available from the corresponding author on reasonable request after signing a data transfer agreement with the receiving institution to ensure the protection of personal information.
