# Supplementary figures and images for: Leveraging Machine Learning to Identify Subgroups of Misclassified Patients in the Emergency Department: Multicenter Proof-of-Concept Study
Source: J Med Internet Res. 2024 Dec 31;26:e56382. doi: 10.2196/56382 (PMC11733519; doi:10.2196/56382)

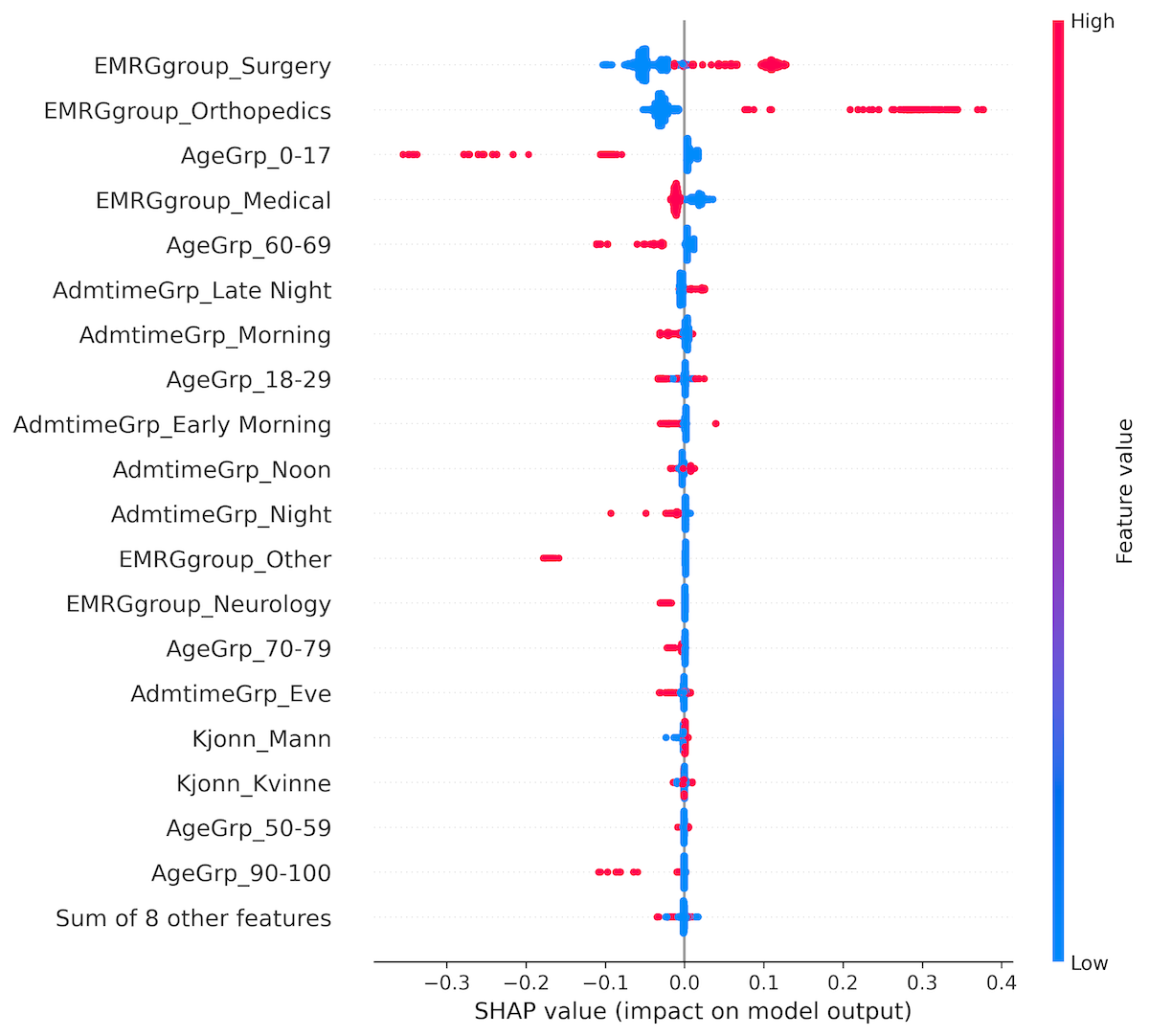

Supplement: Multimedia Appendix 1 [file jmir_v26i1e56382_app1.png]

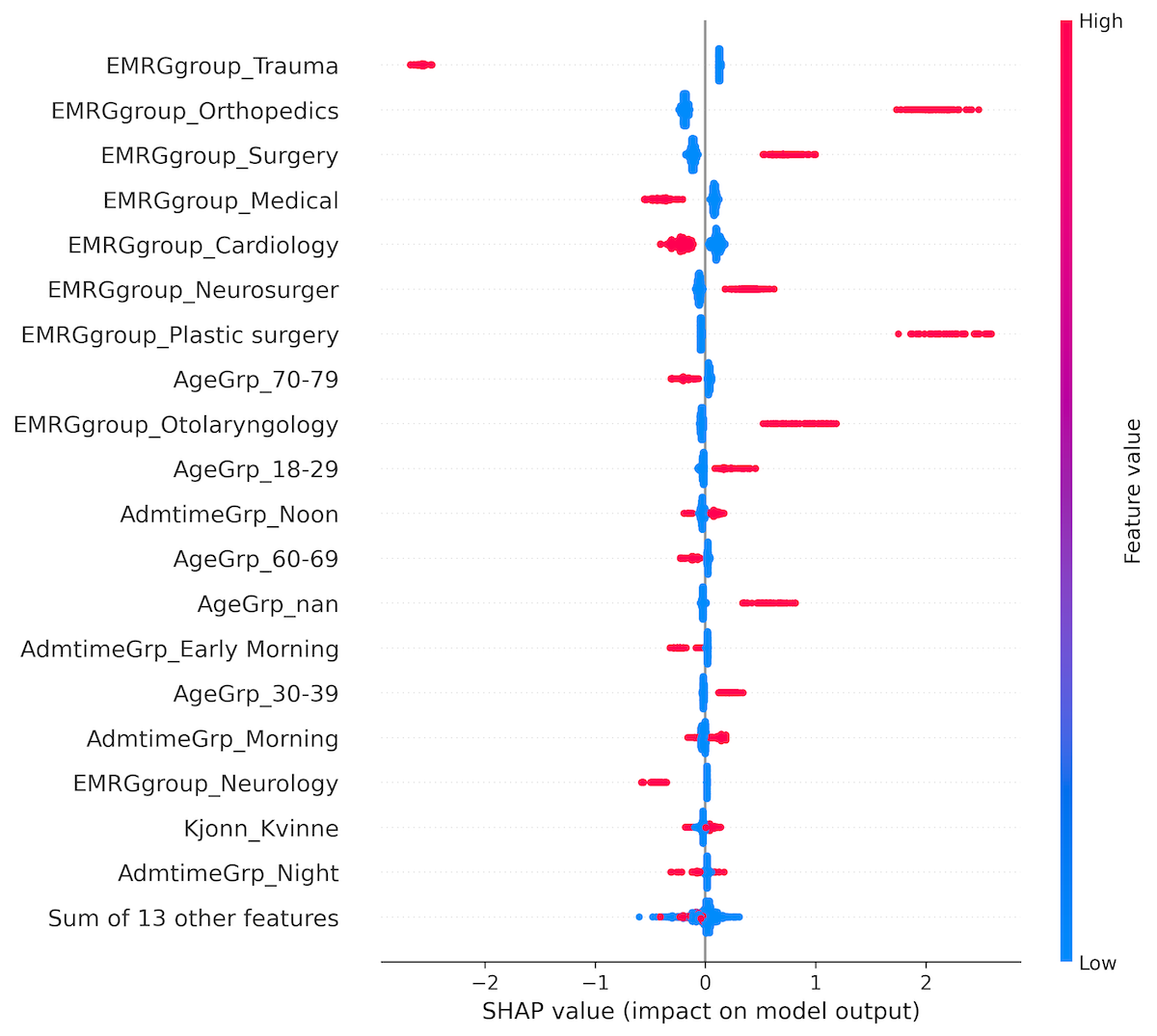

Supplement: Multimedia Appendix 2 [file jmir_v26i1e56382_app2.png]
