# Supplementary material for: Leveraging Machine Learning to Identify Subgroups of Misclassified Patients in the Emergency Department: Multicenter Proof-of-Concept Study
Source: J Med Internet Res. 2024 Dec 31;26:e56382. doi: 10.2196/56382 (PMC11733519; doi:10.2196/56382)
Supplement: Multimedia Appendix 3 [file jmir_v26i1e56382_app3.docx]

# Supplementary material – results for the overtriage models

For overtriage, the most important features in the model were the clinical assignment categories (EMRGgroup), time of day of admission, and patient age. (Suppl. fig. 1). This was again consistent for both the Bergen and Trondheim datasets, with similar relative SHAP-values for each feature in both datasets. Clinical referral department was the most important feature in both datasets.


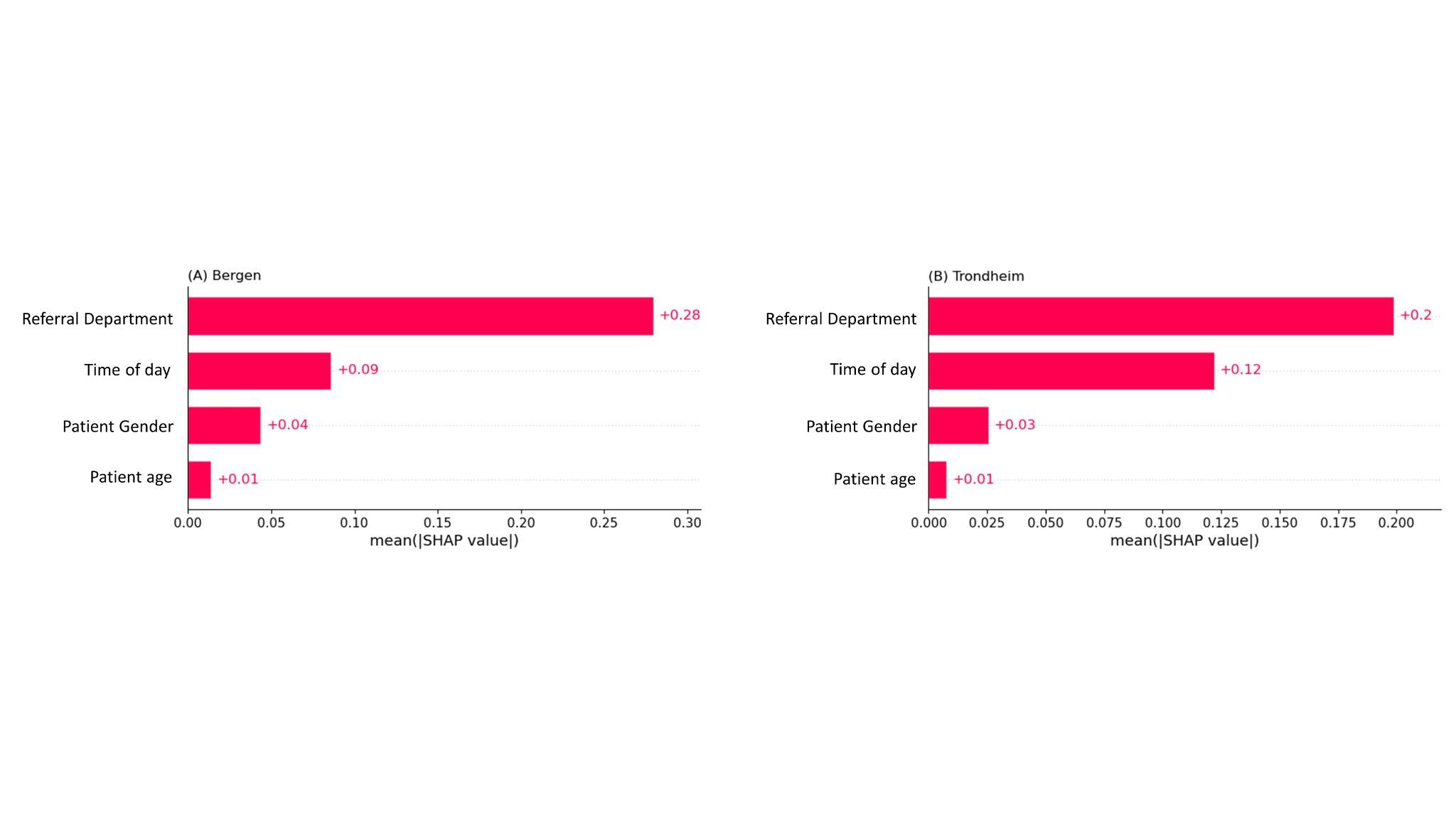


Suppl. figure 1: Mean SHAP-values for overtriage in the Bergen (A) and Trondheim (B) dataset

In the Bergen dataset, the otolaryngology clinical assignment category (Suppl. fig. 2A) and morning admission time (Suppl. fig. 3A) showed a trend towards higher SHAP-values, indicating a higher probability of overtriage, while the “trauma” clinical assignment category shows a shift towards lower SHAP-values, indicating a higher probability of correct triage. Women had higher SHAP-values than men and were more likely to be overtriaged. (Suppl. fig. 4A)


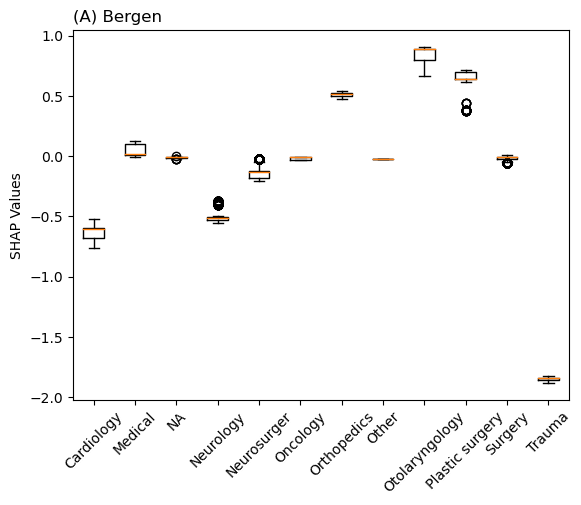

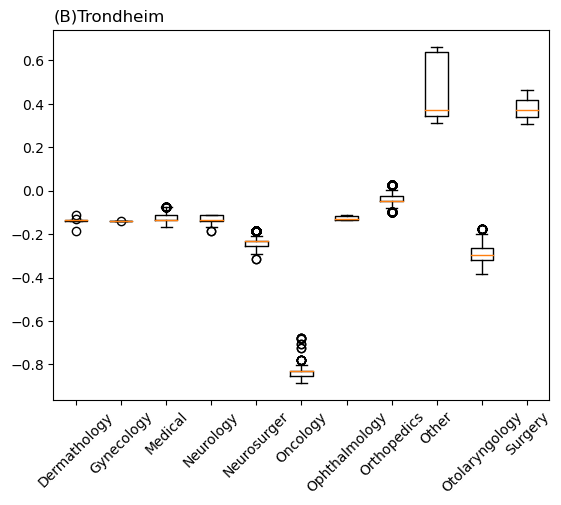


Suppl. figure 2: SHAP-values for overtriage by clinical assignment category in the Bergen (A) and Trondheim (B) dataset


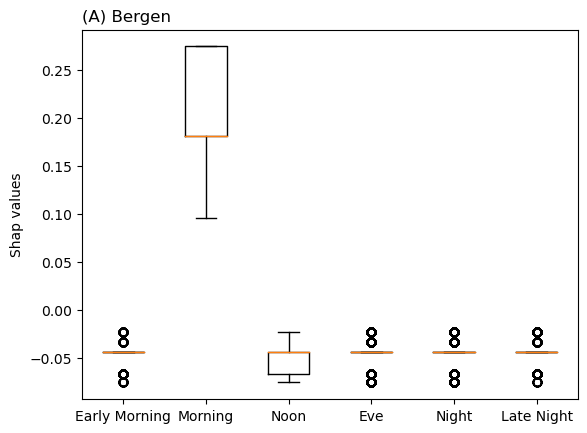

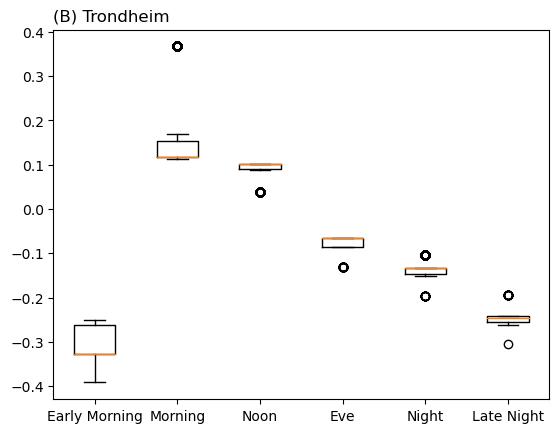


Suppl. figure 3: SHAP-values for overtriage by time of admission in the Bergen (A) and Trondheim (B) dataset


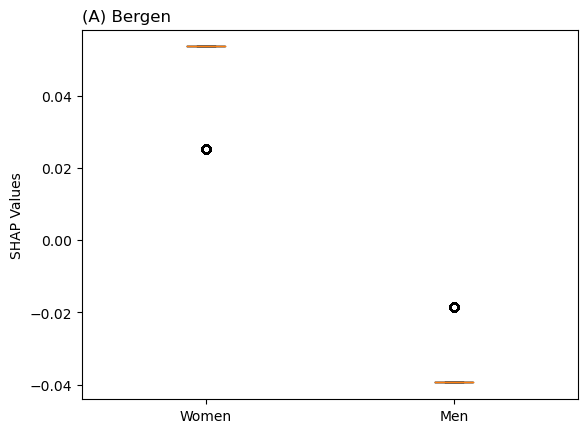

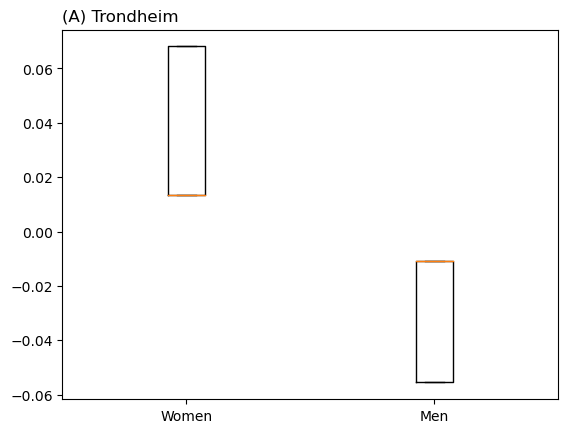


Suppl. figure 4: SHAP-values for overtriage by patient gender in the Bergen (A) and Trondheim (B) dataset

In the Trondheim dataset, we observe the “Surgery” clinical assignment category and other “unspecified” clinical assignment categories (Suppl. fig. 2B) have higher SHAP-values, indicating a higher probability of overtriage, while “Oncology” clinical assignment category and admission times at “late night” or “early morning” (Suppl. fig. 3B) show a shift towards lower SHAP-values, indicating a higher probability of correct triage. Women have a shift towards higher SHAP-values, indicating a higher probability of overtriage, while men have a shift towards lower SHAP-values, indicating a higher probability of correct triage. (Suppl. fig. 4B)
